# Lessons from Model Organisms: Phenotypic Robustness and Missing Heritability in Complex Disease

**DOI:** 10.1371/journal.pgen.1003041

**Published:** 2012-11-15

**Authors:** Christine Queitsch, Keisha D. Carlson, Santhosh Girirajan

**Affiliations:** Department of Genome Sciences, University of Washington, Seattle, Washington, United States of America; Baylor College of Medicine, United States of America

## Abstract

Genetically tractable model organisms from phages to mice have taught us invaluable lessons about fundamental biological processes and disease-causing mutations. Owing to technological and computational advances, human biology and the causes of human diseases have become accessible as never before. Progress in identifying genetic determinants for human diseases has been most remarkable for Mendelian traits. In contrast, identifying genetic determinants for complex diseases such as diabetes, cancer, and cardiovascular and neurological diseases has remained challenging, despite the fact that these diseases cluster in families. Hundreds of variants associated with complex diseases have been found in genome-wide association studies (GWAS), yet most of these variants explain only a modest amount of the observed heritability, a phenomenon known as “missing heritability.” The missing heritability has been attributed to many factors, mainly inadequacies in genotyping and phenotyping. We argue that lessons learned about complex traits in model organisms offer an alternative explanation for missing heritability in humans. In diverse model organisms, phenotypic robustness differs among individuals, and those with decreased robustness show increased penetrance of mutations and express previously cryptic genetic variation. We propose that phenotypic robustness also differs among humans and that individuals with lower robustness will be more responsive to genetic and environmental perturbations and hence susceptible to disease. Phenotypic robustness is a quantitative trait that can be accurately measured in model organisms, but not as yet in humans. We propose feasible approaches to measure robustness in large human populations, proof-of-principle experiments for robustness markers in model organisms, and a new GWAS design that takes differences in robustness into account.

## Introduction

Complex diseases such as diabetes, cancer, and cardiovascular and neurological diseases are the predominant causes of morbidity and mortality in the developed world. As they tend to cluster in families, these diseases are thought to involve genetic factors in addition to environmental ones. Although genome-wide association studies (GWAS) have identified hundreds of common variants associated with complex diseases, and although susceptibility loci have been reported for many disorders, the overall genetic risk explained by these loci remains modest. Thus, only a small proportion of heritability (proportion of phenotypic variance explained by genetic variants) has been accounted for [1], [2]. This discrepancy, termed “missing heritability,” has been attributed to many factors. First, rare variants of large effect size (odds ratio >2) may account for some of the unexplained genetic risk, as observed in neuropsychiatric disorders such as autism, schizophrenia, and developmental delay [1]–[4]. By their nature, rare variants are difficult to detect and to associate with phenotype using statistics. Second, highly repetitive structural and sequence variants have remained inaccessible to large-scale genotyping [1], [2], [5]. Third, heritability estimates may be artificially inflated due to interactions between genes, to shared environments in families, and to imprecise diagnoses of complex disorders [1], [2], [5], [6]. Consequently, current research addresses the problem of missing heritability with more comprehensive genotyping of genetic variants in statistically well-powered cohorts of individuals that are better characterized for disease phenotypes, genetic background, and environmental exposure [1], [2]. This approach is rooted in the prevalent hypothesis that some combination of rare variants of large effect, common variants of small effect, and environmental factors will translate into disease [1],[2],[5].

An alternative view posits that cryptic genetic variation accounts for a substantial fraction of disease-associated risk [7]. In robust individuals, cryptic genetic variation will not contribute to disease and will elude detection by GWAS. In contrast, in individuals with decreased overall phenotypic robustness, formerly cryptic genetic variants will contribute to disease, and disease-related variants will increase in penetrance, resulting in increased heritability. This hypothesis draws on findings from diverse model organisms including yeast, worms, flies, plants, and fish: Decreased phenotypic robustness significantly increases heritability of complex traits due to revealed, formerly cryptic genetic variation and increased penetrance of genetic variants [8]–[16]. In this review, we describe the causes and consequences of decreased phenotypic robustness in model organisms, relate these findings to complex disease phenotypes, and propose an alternative GWAS approach that accounts for differences in robustness among humans.

## What Is Phenotypic Robustness?

Phenotypic robustness is the ability of a given genotype to produce a constant phenotype, even when the organism is faced with genetic or environmental perturbations. The remarkable phenotypic robustness of wild-type organisms is commonly attributed to features of the underlying genetic networks, such as modularity, feedback loops, gene redundancy, connectivity, degeneracy, and the presence of activity-modulating microRNAs [9], [11], [17]–[33]. In model organisms, targeted perturbation of any of these features can decrease phenotypic robustness and release cryptic genetic variation [8], [10], [14], [15], [17], [24]–[26], [32], [34], [35].

Phenotypic robustness is a measurable quantitative trait. Traditionally, robustness of individuals has been measured as the degree of symmetry in morphological features [36]. A high degree of symmetry is thought to be associated with high fitness and even with the perception of beauty for human faces [37]–[39]. In most organisms, objective and high-throughput analysis of symmetry is complicated by the complexity of morphological features and their profound changes throughout development. Another measure of robustness is the degree of accuracy with which a genotype produces a particular phenotype across many isogenic siblings [36]. By this measure, phenotypic robustness, like any other quantitative trait, shows a distribution among genetically divergent individuals of a species and can be mapped to distinct genetic loci [13]. The ability to buffer mutations can vary among isogenic individuals, suggesting that nongenetic mechanisms significantly affect robustness [15], . These nongenetic robustness determinants will elude genetic approaches. None of the robustness measures that have been used in model organisms are applicable in humans; however, they have proven useful to identify master regulators or network hubs that may contribute to robustness in humans.

The best-characterized master regulator of robustness is the molecular chaperone HSP90 [8]–[10], [12]–[16], [21], [34], [40]–[43]. HSP90 assists the proper folding and function of many key enzymes and transcription factors that govern growth and development [43]. The chaperone is essential in eukaryotes, is evolutionarily conserved, is highly connected, and plays a crucial role in integrating environmental signals [43]. HSP90's function is of even greater importance under environmental stress that compromises protein folding [43]. In genetically divergent plant, fly, yeast, and fish populations, HSP90 inhibition significantly increases heritability due to increased penetrance of known genetic variants and revealed cryptic genetic variants [8], [10], [14], [34]. In worms, naturally varying HSP90 levels predict mutation penetrance: lower HSP90 levels result in greater penetrance of mutations [15], [16]. In plants, yeast, and flies, HSP90-dependent variants are common in natural strains and often affect complex traits [12], [13], [34], [44].

## Decreased Phenotypic Robustness Is Associated with Genome Instability

**Figure 1:**
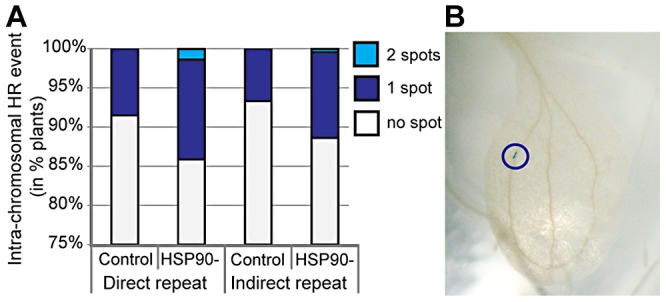
HSP90 inhibition increases homologous recombination in plants. (A) Transgenic plants carrying reporter constructs that monitor somatic homologous recombination (HR) events [48] were grown with and without HSP90 inhibition. HR restores a functional GUS gene, allowing for detection of somatic HR events. Intra-chromosomal HR events were significantly increased in plants grown with the HSP90 inhibitor geldanamycin for direct and indirect repeat reporter constructs (Poisson regression *p* = 0.0078). A total of 925 seedlings were analyzed with 381 seedlings for the direct repeat reporter line and 544 for the indirect repeat reporter line. (B) Example of somatic HR event in plant leaf (blue circle around GUS spot) (unpublished data by K. Carlson, A. Nuttle, and C. Queitsch).

Loss of robustness may be associated with an increased mutation rate. In flies, HSP90 inhibition increases transposon transcription and mobility [40], [42]. In human cells, HSP90 inhibition compromises repair of DNA damage in response to radiation [45] and increases the mutation rate of microsatellites [46]. In yeast, severe HSP90 inhibition induces aneuploidy [47]. In plants, HSP90 inhibition increases the rate of homologous recombination (Figure 1). HSP90 inhibition appears to interfere broadly with genome stability by affecting transposon silencing, DNA repair, microsatellite stability, chromosome segregation, and homologous recombination. Given the extent of standing variation that responds to HSP90 inhibition [12], [13], [34], [44], newly arising mutations probably play a minor role in HSP90-dependent phenotypes, yet genome instability may be a hallmark of decreased robustness. These HSP90-specific results are consistent with stress-induced increases in mutation rate in bacteria, yeast, and plants [47]–[52]. Environmental stress also decreases robustness in diverse organisms, supporting an association of robustness with genome stability [8], [10], [14], [53], [54].

HSP90 is only one of several “robustness” master regulators. In yeast, 60–300 genes, all network hubs, have been identified for which deletion of any one decreases robustness [24]. In worms, decreased function of highly connected hub genes enhances the phenotypic consequences of decreased function in many other genes [35]. It remains untested how many of these yeast and worm network hubs reveal cryptic genetic variation in divergent populations or decrease genome stability when perturbed. However, effects on genome instability seem likely as both worm and yeast network hub genes are strongly enriched for chromatin regulators [24], [35]. In plants, a novel master regulator of robustness has been found, in which loss of function decreases phenotypic robustness and releases cryptic genetic variation [55]. Consistent with an association of robustness and genome stability, inhibition of this master regulator increases transposon mobility [56].

## Release of Cryptic Genetic Variation May Contribute to Rise of Complex Diseases

While both rare variants of large effect and many common variants of small effect contribute to complex diseases [5], neither model satisfactorily explains the significant rise of complex diseases [5], [7]. In the last century, dramatic changes in lifestyle and environment included diet changes, refrigeration, departure from natural circadian rhythm through artificial lighting, modern hygiene, and urban living, to name a few [7], [57]–[59]. In particular, changes in diet and refrigeration have led to the fast evolution and changing composition of the human gut microbiome [60]–[66]. Greg Gibson has suggested that these environmental perturbations may alter the genetic contributions to phenotype by revealing cryptic genetic variation, especially among individuals with reduced phenotypic robustness [5], [7].

Gibson's hypothesis that cryptic genetic variation contributes to disease susceptibility is supported by the properties of disease risk variants and their distribution among human populations. A surprising number of the single nucleotide polymorphisms (SNPs) associated with disease risk are ancestral, indicating that the protective variant arose in the human lineage. Thus, disease susceptibility cannot be easily explained by acquisition of deleterious mutations in the human lineage. Further, disease risk alleles vary dramatically in frequency and effect size in human populations, indicating extensive population structure and the importance of environmental factors for developing disease [7].

## Features of Certain Complex Diseases Are Consistent with Decreased Robustness

As common SNPs fail to confer significant disease risk for disorders such as autism, schizophrenia, and mental retardation, rare variants of potentially large effect have been examined [1], [2]. The human genome contains regions that are predisposed to copy number variation (CNV) due to their repeated architecture [3]. Several of these rare, recurrent CNVs are associated with schizophrenia, autism, cardiac and renal anomalies, epilepsy, obesity, diabetes, and mental retardation [3], [4], [67]–[72]. However, many of these CNVs are also found in control populations and unaffected family members. Moreover, the same CNV can be associated with a large spectrum of disorders [3], [4]. For example, del17q12 is associated with renal cysts, maturity-onset diabetes, developmental delay, brain malformations, seizures, schizophrenia, and autism [3], [4]. This variability in expressivity is thought to be due to additional rare events: the classical genetic modifier hypothesis [3], [4], [68], [69], [73]. Alternatively, such CNV lesions may not be causal but rather reflect decreased genome stability [74] and decreased robustness.

If decreased robustness correlates with or causes genome instability, patients with complex diseases should carry a higher burden of CNVs. This burden (or mutational load) may be inherited or may arise *de novo* through environmental stress in early development. Indeed, schizophrenia patients show a significantly higher global burden of rare CNVs [72], [75]. Most importantly, private CNVs—that is, CNVs specific to a particular individual—are highly enriched in schizophrenia patients [75]. A similar increase of CNV burden is found in autism patients [73]. Patients with the recurrent CNV on chromosome 16p12.1, which is associated with severe developmental delay, are also more likely to carry additional CNVs than matched controls [69]. Moreover, patients with second CNV hits—possibly less robust individuals—show distinct and more severe clinical features [69]. Consistent with decreased robustness, the facial symmetry of some patients is visibly perturbed [69]. In nine other genomic disorders, additional CNV hits occur more frequently in patients than controls [69]. This enrichment of CNV hits in patients is particularly strong for disorders with variable penetrance and expressivity [3], [4], [69]. In multiplex autism families (families with multiple occurrences), CNVs in affected siblings are fourfold enriched compared to unaffected siblings [76]. This observation led to the hypothesis that multiplex autism is due to an inherited predisposition in addition to other co-occurring mutations, including CNVs [3], [76]. We speculate that this predisposition may be decreased robustness.

The observation of additional, mostly private CNVs in patients is consistent with the existence of an extraordinarily large number of distinct genetic modifiers leading to disease. While certainly possible, this explanation is not consistent with our basic knowledge of genetic networks and their robustness to mutations. In yeast and worms, for which systematic analyses of single and double mutant phenotypes have been conducted, most loss-of-function mutations, even if combined, do not show a phenotype unless they occur in network hubs or master regulators such as HSP90 [24], [35], [77]–[79]. The number of network hubs in humans is large but not infinitely large. Under our hypothesis, the increased CNV burden is an expression of a generally less robust and therefore sensitized background rather than a cause of disease. This explanation is consistent with the fact that the same CNV can be associated with different disorders. The different expressivity of a particular disorder may be due to different degrees of robustness loss or different revealed, formerly cryptic genetic variants in patients. In short, we propose that phenotypic robustness differs among humans as it does in model organisms and that those with lower robustness will be more susceptible to genetic and environmental perturbations and hence disease. This proposal is akin to assertions from physicians of generations past that certain people have a robust constitution whereas others have weak ones.

## How Can We Assess Robustness in Humans to Increase the Heritability of Complex Diseases?

**Figure 2:**
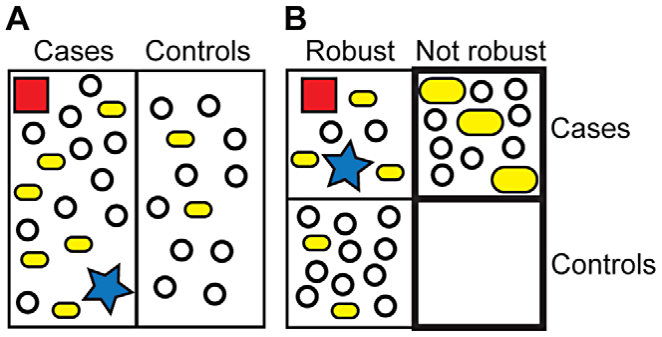
Current and suggested GWAS approaches. (A) Current approach. GWAS identify variants that are overrepresented in cases. Rare variants of large effect (red square, blue star) may escape detection, thereby contributing to missing heritability. Common variants that are overrepresented in cases (small yellow bar, 6 versus 2) do not contribute strongly to disease risk. A cryptic disease-related variant does not show significant overrepresentation in cases (open circle). (B) Suggested approach. Individuals are first analyzed for phenotypic robustness (bold box) and then for variants associated with disease. Rare variants of large effect will be enriched in robust cases, although they may also be present in nonrobust cases. Variants that are overrepresented in all cases (robust, nonrobust) will show higher penetrance in nonrobust individuals (large yellow bars). The formerly cryptic, disease-related variant (open circle) is significantly enriched in nonrobust cases versus nonrobust controls (and robust cases) and can therefore be identified. Together, heritability significantly increases. The formerly cryptic genetic variant and higher penetrance variant can be thought of as “disease-specifiers” as they determine the specific disease phenotype of individuals carrying them. Note symbols represent highly simplified frequencies of specific variant in indicated groups and not individuals carrying certain variants.

Currently, researchers attempt to identify unifying patterns of genetic variants, lifestyle choices, and environmental factors in affected individuals (cases, Figure 2). In contrast, we would like to assess all individuals first for their level of phenotypic robustness (robust versus not robust, Figure 2). Individuals with significantly decreased robustness would then be analyzed for genetic variants associated with disease. This analysis will identify formerly cryptic variants that were unknown to influence disease risk. In addition, previously identified predisposing variants will increase in penetrance because healthy individuals carrying these variants will be robust and therefore not contribute to associations among the group with decreased robustness. In addition, this analysis will facilitate the identification of rare causal variants of large effect, which should be enriched in robust but affected individuals. Increased penetrance of common variants together with revealed, formerly cryptic variants and causative rare alleles of large effect will significantly increase heritability for at least some complex diseases.

This approach hinges critically on the identification of reliable markers for phenotypic robustness that can be readily assessed in large human populations. The functionality of master regulators such as HSP90 could potentially provide such a robustness measure. In yeast, however, the group of master regulators that affect robustness is large and functionally diverse (about 1%–5% of all nonessential genes) [24]. Assaying the diverse functions of hundreds of proteins is not a suitable high-throughput test.

Because decreased robustness correlates with and produces genome instability at several levels—an increase in microsatellite mutations, transposon mobility, recombination rates, base-substitution mutation rate, and large duplications and deletions—we suggest that these different genome instability events can serve as readouts for decreased robustness. Recent technological advances have made most of these features easily accessible in large populations of humans and model organisms. The individual events may be rare, as observed for CNV variation; hence, they need to be investigated on a genome-wide scale. As microsatellites show by far the greatest mutation rate [80], somatic microsatellite variation may be the most sensitive robustness marker. In fact, microsatellite variation has a long history as a marker for deficient DNA repair in certain cancers [81], [82]. At this point, however, assessment of microsatellite variation requires high-quality, expensive Sanger-sequencing. Neither the more cost-effective next-generation sequencing nor array-based genotyping can accurately determine variation of small microsatellites, but given the pace of current technology development, this technical hurdle should soon disappear.

Before investing in human studies, the suitability of molecular robustness markers is easily testable in model organisms. We envision the following proof-of-principle experiments: First, a diverse wild-type population, ideally freshly collected, is phenotyped for robustness, using traditional robustness measures such as symmetry or quantitative trait variation in genetically identical offspring (such as exists in plants and worms). We expect a distribution of robustness. Second, the least robust and most robust individuals are assessed for genome instability events at several levels. We expect to observe a higher frequency of these events in less robust individuals. If a significant correlation between a traditional robustness measure and any genome instability event is found, this type of genome instability is a robustness marker that is applicable to humans. Third, if our hypothesis is correct, less robust individuals should be more susceptible to environmental stresses and will show higher expressivity of mutations and genetic variation. This assumption can be tested by exposing the least robust and most robust individuals to environmental stress and mutagenesis.

If genome instability events fail to predict robustness, alternatives exist. For humans, DNA-, RNA-, or cell-based assays are preferable, as the necessary material can be obtained with relative ease. Indeed, several cell-based, high-throughput assays for somatic mutations already exist for humans [83]. In principle, these assays monitor allele loss resulting in an altered phenotypic output such as fluorescence. Another, potentially more promising, cell-based approach for determining robustness in humans would be to assess cell population variance for a given individual in gene expression, genome methylation, or chromatin states. Cell population variance in shape and other morphological features could also serve as a robustness marker [24]. In yeast, this approach identified robustness master regulators by calculating the variance of 70 phenotypic traits among individual cells stained for nuclei, actin, and a cell wall marker [24]. Given this arsenal of possible robustness measures, high-throughput molecular or cell-based robustness markers seem feasible in the near future.

Our hypothesis is testable even in the absence of human robustness markers. If decreased robustness predisposes to complex diseases, we can test whether individuals suffering from one complex disease are more likely to suffer from another, as we would predict. Another test would compare variants between cohorts of patients suffering from two different complex diseases. This comparison would filter out shared variants that are not causative and possibly related to decreased robustness and reveal statistically enriched causative variants that are specific to each disease.

Our hypothesis that robustness differences among individuals contribute to the missing heritability of disease is akin to prior propositions that epistasis (i.e., genetic interactions) accounts for the missing heritability [5], [6]. Epistatically interacting loci could certainly be detected through GWAS, yet this will require very large sample sizes to find sufficient individuals of each genotype combination. In contrast, our approach reduces the intractable complexity of possible genetic interactions to robustness, which we propose is a universal disease and trait modifier that can be feasibly measured in large human populations. Furthermore, not all instances of disease may involve genetic interactions; some may arise from interactions of risk alleles with nongenetic mechanisms or environmental factors. Unlike traditional GWAS, our approach might capture these instances because robustness differences can arise through nongenetic mechanisms and environmental perturbations. Taking robustness into account has the potential to free us from disentangling the multitude of factors contributing to specific instances of disease. If successful, this approach might render complex disease more deterministic and predictable, allowing us to better identify the contributing lifestyle choices and environmental exposures and ultimately decrease the severity and incidence of these devastating diseases.
